# The Intimate Structure of the Secreting Glands

**Published:** 1840-04

**Authors:** 


ART. III.—*The Intimate Structure of the Secreting Glands, by* JOHN MÜLLER, M.D., *with the subsequent discoveries of other Authors.* By SAMUEL SOLLY, F.R.S., Lecturer on Physiology, and on Comparative Anatomy, at St. Thomas's Hospital, &c. &c. With four plates, and woodcuts.—*London,* 1839. 8vo, pp. 166.

IN pursuance of his laudable endeavour to excite attention in this country to the great truth that physiology can only be pursued with the certainty of success when it is based on comparative anatomy, Mr. Solly has brought the substance of Müller's splendid monograph on the glandular system within the means of the English student; and he has incorporated with it several contemporaneous and subsequent discoveries made by other enquirers. Thus we find in this little volume the analysis of Dr. Boehm's observations on the intestinal glands, which appeared in our own pages; the description given by Mr. Morgan of the curious mammary apparatus of the kangaroo; a summary of Mr. Kiernan's researches on the minute anatomy of the liver; and notices of the points in which Sir A. Cooper differs from Müller on the anatomy of the testicle. Besides these larger additions, we have allusions to Hunter's labours, of which Müller knew little or nothing, and which had anticipated him (as well as almost every one else) in many points: and of some recent enquiries on the part of Mr. Owen. We could have wished that the long extract from Mr. Corfe's book on the kidney had not been inserted. The doctrines, both anatomical and physiological, which it contains, are so strange, that they must be received with suspicion; and, although Mr. S. speaks of having seen Mr. C.'s preparations, he does not appear satisfied of the accuracy of the description of them which he has quoted.

In an appendix we find a full account of Dr. Sprott Boyd's observations on the structure of the mucous membrane of the stomach, and of the researches of Bischoff and Purkinje on the same subject; as well as a summary of Dr. John Davy's late description of the male organs of some cartilaginous fishes.

From this summary of the contents of Mr. Solly's volume, it will, we think, appear that it is one well worthy of the student's acquaintance. The analysis of Müller's work is, on the whole, well executed, and the other selections are made with judgment. The chief fault we have to find is, that the materials are not very well blended together. The student will in vain look elsewhere, however, for the same amount of information in so narrow a compass and at so cheap a rate. We must not omit to notice the four lithographs, which contain upwards of seventy subjects, copied chiefly from Müller's folio plates, and well drawn by Mr. Solly.

